# The progress of PET/MRI in clinical management of patients with pancreatic malignant lesions

**DOI:** 10.3389/fonc.2023.920896

**Published:** 2023-04-28

**Authors:** Jindan Li, Chaojiang Fu, Sheng Zhao, Yongzhu Pu, Fake Yang, Shuguang Zeng, Conghui Yang, Hongqiang Gao, Long Chen

**Affiliations:** ^1^ Department of PET-CT/MR Center, Yunnan Cancer Hospital, The Third Affiliated Hospital of Kunming Medical University, Kunming, Yunnan, China; ^2^ Department of Emergency, Yunnan Cancer Hospital, The Third Affiliated Hospital of Kunming Medical University, Cancer Center of Yunnan Province, Kunming, Yunnan, China; ^3^ Department of Information Center, Yunnan Cancer Hospital, The Third Affiliated Hospital of Kunming Medical Cancer Center of Yunnan Province, Kunming, Yunnan, China; ^4^ Department of Hepatobiliary Surgery, The First People’s Hospital of Kunming City & Ganmei Affiliated Hospital of Kunming Medical University, Kunming, China

**Keywords:** pancreatic cancer, PET/MRI, emerging imaging agents, efficacy monitoring, prognosis evaluation

## Abstract

Recently, the morbidity and mortality of pancreatic cancer have been increasing year by year. Because of its deep anatomical location and because most presented patients often suffer from abdominal pain or jaundice, it is difficult to diagnose pancreatic cancer at an early stage, leading to late clinical stage and poor prognosis. integrated positron emission tomography/magnetic resonance imaging (PET/MRI) fusion imaging not only has the characteristics of high resolution and multi-parameter imaging of MRI, but also combines the high sensitivity and the semi-quantitative characteristics of PET. In addition, the continuous development of novel MRI imaging and PET imaging biomarkers provide a unique and precise research direction for future pancreatic cancer research. This review summarizes the value of PET/MRI in the diagnosis, staging, efficacy monitoring, and prognosis evaluation of pancreatic cancer, and prognosis for developing emerging imaging agents and artificial intelligence radiomics in pancreatic cancer.

## 1 Introduction

In recent years, PET/MRI, a new multimodal imaging system that integrates PET and MRI, has attracted a lot of attention. It realizes the simultaneous acquisition of their respective data by two different devices in the same space, and confers the independent functions of each device. It not only combines the high soft-tissue contrast and multi-parameter imaging characteristics of the MRI system, but also combines the high sensitivity of the PET system and data quantification characteristics; there in PET/MRI possesses distinct advantages in reflecting anatomical structure morphology and physiological, functional information (1)

Pancreatic cancer is the seventh leading cause of cancer-related death globally, and the incidence is increasing yearly worldwide, especially the incidence of pancreatic ductal adenocarcinoma (PDAC) is increasing at a rate of 0.5% to 1.0% per year (2). More than 430,000 people die each year from the disease, which is projected to become the second leading cause of cancer-related death by 2030. The pancreas is located deep in the abdomen, behind the stomach and in front of the spine, and most patients with pancreatic cancer go to the doctor when they have skin jaundice and abdominal pain, which makes the early diagnosis and detection of the pancreas difficult. Due to the late clinical stage of most patients, the five-year survival rate is nearly 2%-9% (3). Prompt diagnosis and treatment are of great significance to improve the prognosis of pancreatic cancer patients. Studies have shown that pancreatic cancer patients with early detection have an improved prognosis than symptomatic patients, and there is a sufficient early detection window (4). Although carbohydrate antigen 19-9 (CA19-9) is the most critical, serum biomarker for pancreatic cancer, the sensitivity and specificity of CA19-9 for the early diagnosis of pancreatic cancer are 0.748 and 0.782, respectively (5); therein, this biomarker is not recommended by many experts for timely diagnosis of pancreatic cancer. No other tumor-specific markers recommended for early diagnosis (6). Many international guidelines point out that multi detector computed tomography (MDCT) is the most extensive and effective tool for imaging pancreatic cancer patients. An expansive meta-analysis comparing various imaging modalities for the diagnosis of pancreatic cancer (7) found that CTs combined sensitivity and specificity of CT and were 89% and 90%, respectively, comparable to MRI. However, the value of MRI is very important for the differential diagnosis of patients with pancreatic isodense lesions on CT, uncertain lesions of liver on CT, impaired renal function or iodine radiography allergy. Especially in these special cases, such as tumors with a diameter of less than 2cm, and the presence of hypertrophic pancreatic head or local fat infiltration in the parenchyma, most experts agree that MRI is more useful than CT (8).

In addition, although merely a handful of studies have compared contrast-enhanced positron emission computed tomography (CE-PET/CT) and PET/MRI, they still show that PET/MRI has the advantages of high sensitivity and specificity (9, 10).

As a new device, PET/MRI is under dynamic research in the clinical management of patients with pancreatic cancer. This article reviews the value of PET/MRI in the clinical management of patients with pancreatic malignant lesions.

## 2 Development and significant advantages of PET/MRI

PET and MRI are well-established techniques used in preclinical research and clinical trials, respectively. Combining PET and MRI was first proposed by Jahren in the late 1990s; this combination generates moreover comprehensive, temporally-matched data given the usage of both imaging modalities (11, 12). However, integrating the two technologies into one machine has perpetually remained a huge challenge hindering the development of PET/MRI. These technical challenges have now been overcome, successfully demonstrating the great potential of PET/MRI scanners in preclinical applications (13–15). In terms of clinical applications, over the past 20 years, as PET/MRI has continued to solve wide-ranging clinical problems, much available hardware has been developed (16). Currently, whole-body integrated PET/MRI systems for clinical use have been launched. Compared with PET/CT, PET/MRI has apparent advantages in guiding the diagnosis and treatment of different diseases.

On the one hand, PET/CT is limited by reasons such as free-breathing, low-dose radiation, and lack of contrast of intravenous contrast agents, so it cannot accurately assess the anatomy of complex lesions. On the other hand, MRI provides an ameliorated, signal-to-noise ratio and contrast noise than CT (17). The multi-parameter imaging of MRI (including fat-suppressed T2-weighted imaging and dynamic T1-weighted fat-suppressed contrast-enhanced images) and multifunctional imaging techniques, such as diffusion-weighted imaging(DWI), magnetic resonance spectroscopy (MRS), and perfusion weighted imaging (PWI) can help identify, localize, and characterize lesions that cannot be otherwise identified by conventional imaging (18). Therefore, PET/MRI not only combines the high soft-tissue contrast and multi-parameter multifunction imaging characteristics of the MRI system, but also combines the high sensitivity and data quantification characteristics of the PET system, which are practical for the diagnosing, grading, staging, restaging, prognosing, and evaluating the efficacies of treatments.

However, PET/CT images need to be acquired sequentially (i.e., low-dose attenuation-corrected CT scans are performed first, followed by the PET scan. Subsequently, the two images are fused, which results in insufficient localization of lesions (19). As a new variant of multimodal fusion imaging technology, PET/MRI integrates two independent imaging technologies, PET and MRI, and achieves accurate data synchronization *via* real-time fusion of anatomy and anatomical function, including that of metabolism and other biochemical processes. The acquisition, allows for more precise anatomical localization of hypermetabolic lesions visualized by PET. PET/MRI also minimizes motion-induced artifacts, especially in the epigastric region via, the physiological gating of acquired data (20). In addition, there is trying to use advanced motion correction algorithms to fully utilize the potential of PET/MRI integration (21). Since the integrated PET/MRI acquisition time is longer than that of PET/CT, more PET data can be collected, reducing noise and improving sensitivity for visualization of subtle lesions.

PET/MRI also significantly reduces radiation dose, and the examination does not generate radiation from the CT portion. Studies have shown that the average dose of 18F-FDG PET/CT is 14 mSv, and the average radiation dose of PET/MRI can be reduced to 7-10 mSv (22). Because some patients undergoing PET/CT require a large number of scans, the reduction in total dose is considerable.

## 3 Clinical features of malignant pancreatic lesions

Among malignant lesions, ductal adenocarcinoma and its variants account for 90% of all pancreatic cancers. Nearly 60%-70% of pancreatic adenocarcinomas occur in the head of the pancreas, and the remainder of growths occur in the body (15%) and tail (15%) (3). The morphology of pancreatic cancers recognized in the World Health Organization Classification of Pancreatic Tumors has different histological features and, possibly because of the different molecular characteristics of these types of pancreatic malignancies, prognoses also vary widely (23).

Pancreatic cancer is a malignant tumor with high degree of malignancy, ease of metastasis, rapid progression, and poor prognosis. Pancreatic cancer has a 5-year survival rate of only 9% and is the seventh leading cause of cancer death in both men and women worldwide (24). The only promising cure for pancreatic cancer is radical surgical resection. However, only 20% of patients have the opportunity for surgical treatment, and more than 50% of pancreatic cancer patients have distant metastases at the time of initial diagnosis. Selecting the most appropriate treatment (e.g., surgical resection, postoperative neoadjuvant therapy, or palliative care) and voiding unnecessary surgery are critical. Also vital are early diagnosis, accurate staging, and timely response evaluation. In these processes, imaging plays an irreplaceable role.

## 4 The value of 18F-FDG PET/MRI in the clinical management of pancreatic malignant lesions

### 4.1 The value of 18F-FDG PET/MRI in the diagnosis of pancreatic cancer

**Figure 1:**
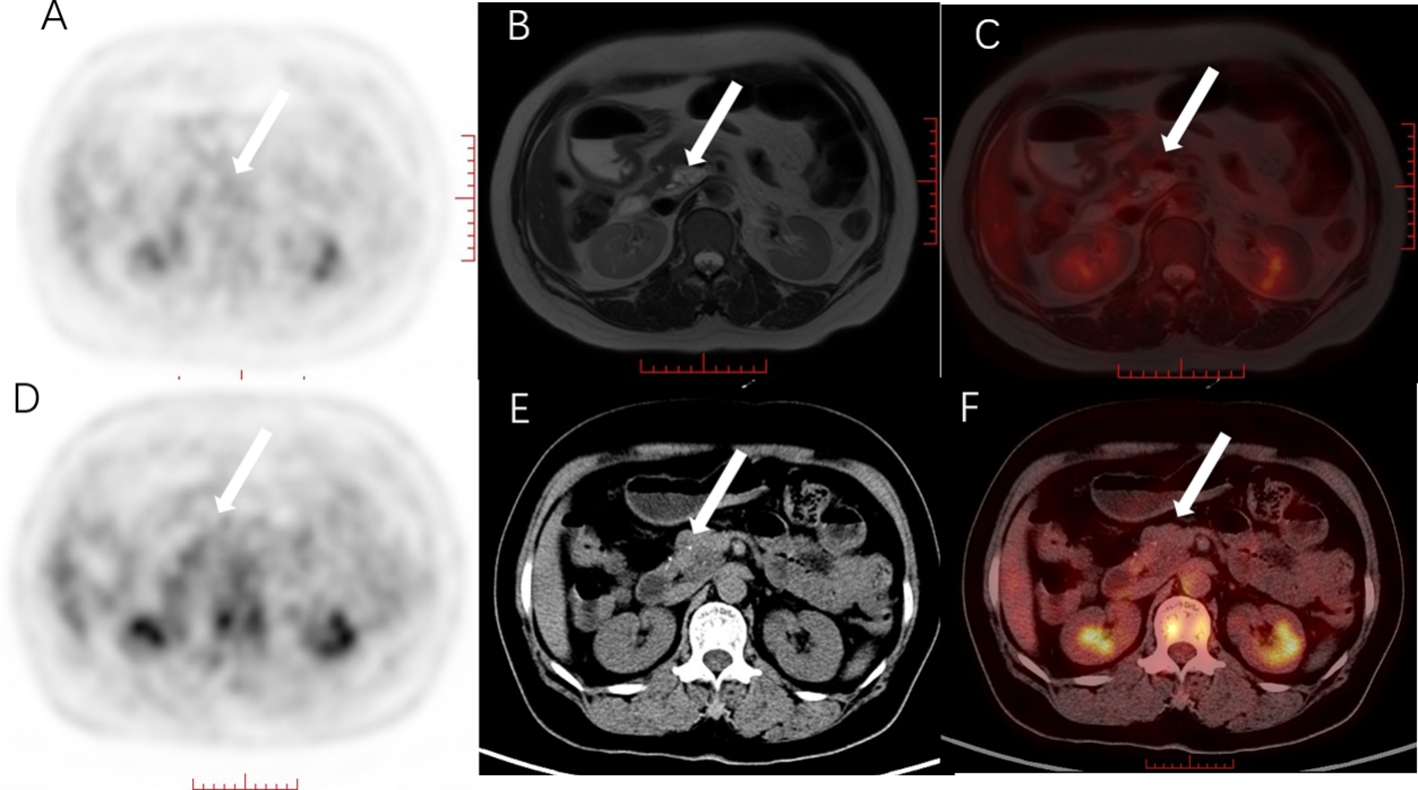
Female, 56 years old, with mucinous cystic tumor of the pancreatic head. **(A)** PET MIP image, no obvious hypermetabolism was found in the pancreas; **(B)** MRI T2WI, T2WI showed heterogeneous and slightly hyperintensive cystic-solid lesions; **(C)** PET/MRI fusion image, there was no metabolism on the fusion image, but A clear display of the cystic and solid components of the lesion is helpful for accurate diagnosis. **(D)** PET MIP image, suspected hypermetabolic foci in the pancreatic head area; **(E)** plain CT scan, the pancreatic head lesion was unevenly low-density, and dot-like high-density can be seen in it; **(F)** PET/CT fusion image, PET/CT fusion images showed no obvious metabolism compared with the pancreatic background. The white arrow shows the location of the lesion. PET/MRI fusion images clearly show the cystic and solid components of the lesions, which is helpful for accurate diagnosis.

The most commonly used clinical diagnostic equipments for pancreatic cancer are CT and MRI. Although CT is the first-line imaging modality for diagnosing pancreatic cancer, both CT and MRI have high sensitivity in detecting pancreatic cancer (96% VS 93.5%). Tumor resectability is superior to MRI, with an accuracy rate of 86.8% vs 78.9% (25). However, with the in-depth research and exploration of pancreatic cancer, PET/MRI also has unique advantages in the diagnosis of pancreatic cancer. Studies by Tatsumi (26) et al. showed that the diagnostic accuracy of fused images of PET and T1WI and T2WI for pancreatic cancer (93.0% and 90.7%, respectively) was better than PET/CT (88.4%), and PET/T1WI was the most efficacious. Studies such as Nagamachi (27) also showed that compared with PET/CT, PET/MRI fusion images had higher accuracy in diagnosing pancreatic cancer (96.6 vs 86.6%) (28). The detection rate of PET/MRI was significantly higher than that of PET/CT for tumor internal structures (such as septa and wall nodules), adjacent blood vessels, common bile duct, main pancreatic duct, and gastrointestinal invasion (see **Figure 1**).

### 4.2 The value of 18F-FDG PET/MRI in the detection of distant metastasis of pancreatic cancer

Pancreatic cancer usually preferentially metastasizes to the liver and peritoneum (29). However, the assessment of liver lesions by PET/CT is often limited due to the high background of FDG uptake by the liver and other inherent technical limitations. Likewise, PET/CT faces serious challenges in detecting minute peritoneal metastases. Integrated PET/MRI can not only capture the advantages of PET functional imaging in M ​​staging but also directly solve the technical limitations of PET/CT for liver evaluation through MRI. The functional and metabolic information provide by PET can also increase the confidence of MRI in diagnosing peritoneal lesions. A meta-analysis conducted by Lee et al. showed that PET/MRI is more advantageous for subtle lesions, including peritoneal lesions.

**Figure 2:**
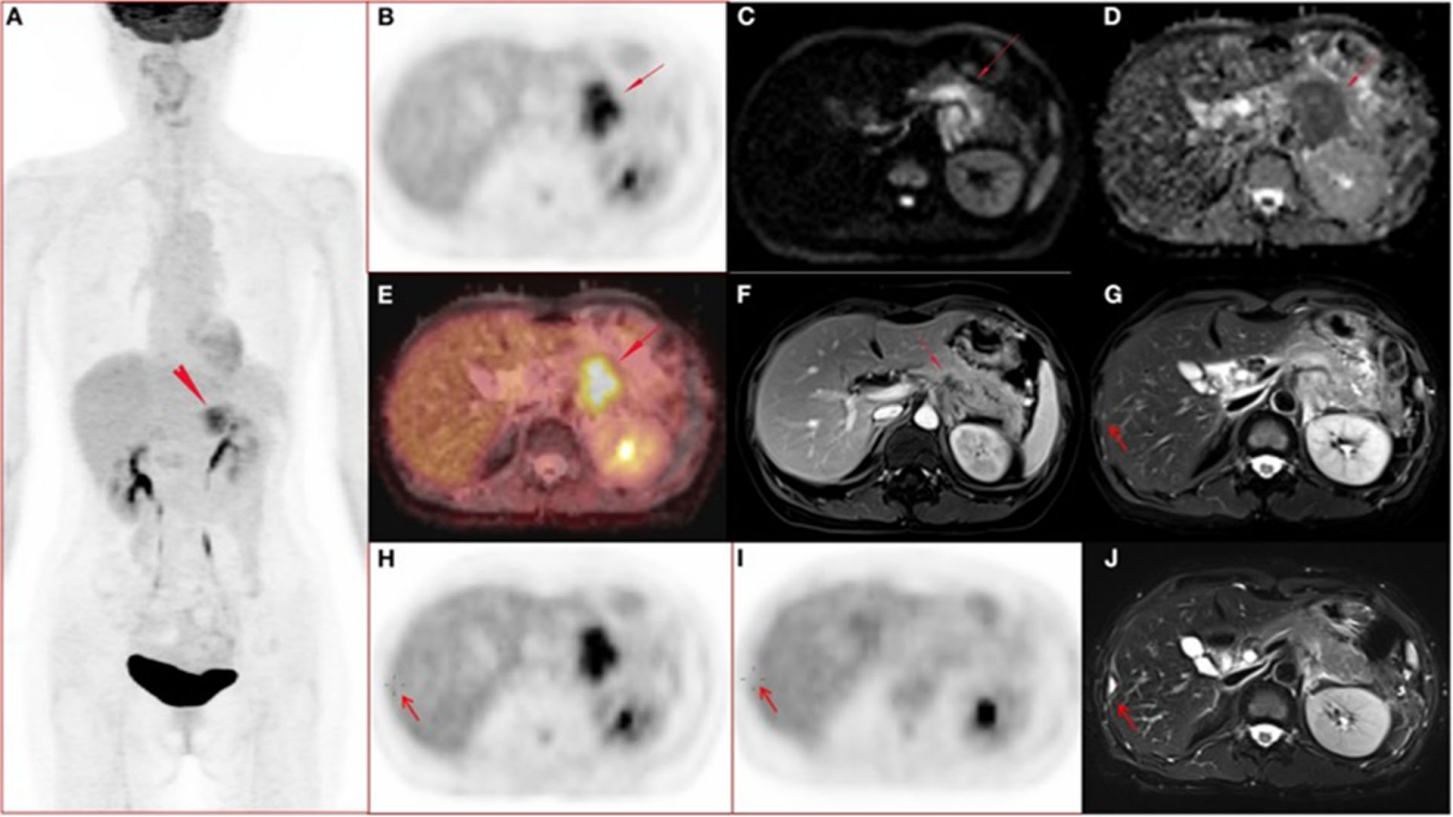
A 51-year-old woman with ductal adenocarcinoma in the body-caudal junction of the pancreas with liver metastases (31). **(A-J)** Whole body PET images; **(A)** maximum intensity projection (MIP); **(B)** transverse abdominal PET images showing increased FDG metabolism in pancreatic body and tail lesions with SUVmax of 7.52, SUVmean of 6.39, and MTV of 17.43 cm3. **(C, D)** Diffusion-weighted imaging (DWI, b = 800) and apparent diffusion coefficient (ADC) showed diffusion-limited lesions in the pancreatic body and tail. **(E)** Fusion image of PET and ADC showing diffusion-restricted lesions with high FDG metabolism. **(F)** Arterial late fat-suppressed (fs) contrast-enhanced (CE) T1-weighted image (T1WI) showing hypovascular disease and dilated main pancreatic duct with a lesion measuring 4.7 cm in maximum diameter. **(G, H)** Right hepatic lobe metastasis (arrow) confirmed by surgery (2 days after initial PET/MRI) and histopathological examination, with mildly hyperintense lesions on T2WI and no FDG uptake on PET images. **(I-J)** 112-day postoperative PET/MRI follow-up showed that the T2WI in the surgical area was markedly hyperintense, and the FDG uptake in PET images was normal.

In addition, using imaging markers such as 18 F-FDG glucose metabolic activity and DWI of PET/MRI to assess the conventional characteristics and quantitative parameters of PET and apparent diffusion coefficient (ADC) can predict the potential factors affecting the metastases of patients with PDAC (30). In the univariate analysis of conventional parameters, the lesion volume in the metastatic group was significantly larger than that in the non-metastatic group, and the PET quantitative parameters [i.e., standardized uptake value (SUV), metabolic tumor volume (MTV), and the peak of total lesion glycolysis (TLG)] were also higher. In the texture feature analysis, four features in the PET image and thirteen features in the ADC map significantly different between the two groups. Multivariate analysis showed that one feature of PET and three features of ADC were significant predictors of PDAC metastasis before treatment. These results suggest that multiple parameters and texture features of the primary tumor in18F-FDG PET/MRI images may be reliable imaging markers for predicting synchronous metastatic disease in PDAC before treatment (**Figure 2**).

In conclusion, PET/MRI offers higher diagnostic accuracy than PET/CT, especially for the diagnosis of distant metastases. More research is needed to confirm the advantages of PET/MRI in pancreatic cancer staging.

### 4.3 The value of 18F-FDG PET/MRI in staging and grading pancreatic cancer

Accurate assessment of pancreatic cancer initial staging, especially N and M staging, and resectability are critical for selecting the most appropriate treatment options (e.g., surgical resection, postoperative neoadjuvant therapy, or palliative care). Among them, surgical resection needs to achieve the effect of obtaining a negative resection margin in order to be an effective treatment. Therefore, preoperative assessment of tumor resectability is also crucial for predicting the prognosis of patients (32). As a systemic examination, PET/CT is undoubtedly a critical imaging examination method for the initial staging and preoperative evaluation of pancreatic cancer (33). However, PET/CT is insufficient for visualization of tumor margins, assessment of adjacent tissue invasion, and detection of lymph node metastases and subtle distant metastases. PET/MRI has high soft-tissue contrast and multiparameter imaging, which are expected to make up for the insufficiency of PET/CT and provide indispensable clinical value for the initial staging and preoperative evaluation of pancreatic cancer (34). In addition, resectability largely depends on the relationship between the tumor and blood vessel for locally advanced pancreatic cancer. For example, patients with a history of mesenteric vascular involvement (such as involvement of the celiac axis, hepatic artery, SMA, portal vein, or superior mesenteric vein) are considered unresectable. With the improvement of treatment levels, resection of pancreas and involved blood vessels is an alternative to palliative treatment for patients with locally-advanced pancreatic cancer. However, vascular resection and locally-advanced pancreatic cancer reconstruction have a high recurrence rate and poor prognosis (35).

**Figure 3:**
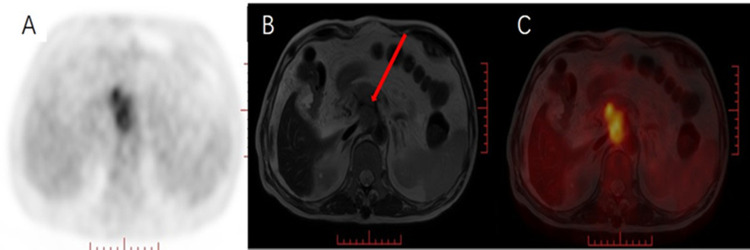
Male, 69 years old, with pancreatic cancer. **(A)** PET MIP image, heterogeneous hypermetabolism was seen in the pancreatic head area, **(B)** MRI T2WI pancreatic head space-occupying boundary was unclear, showing uneven isointensity, **(C)** PET/MRI fusion images of, and, respectively, pancreas Compared with the pancreatic background, the mass in the head area is inhomogeneous and hypermetabolic. Pancreatic head lesions compress the hepatic artery, but the course of the hepatic artery is clearly visible (red arrow).

PET/MRI can clearly depict the relationship among lesions, blood vessels, and metabolic status *via* fusion of multiple sequences of lesion PET imaging and MRI to evaluate the resectability of pancreatic cancer lesions. **Figure 3** shows a patient with pancreatic cancer who underwent PET/CT and MRI examinations before surgery. By fusing the PET/MRI images, it was evident that the pancreatic head lesion compresses the hepatic artery, but the hepatic artery runs clearly. Chen et al. (36) explored the correlation between multiple imaging parameters from PET, DWI and MRS in integrated PET/MRI, including ADCmin, choline level, MTV, TLG and TNM clinical stage of pancreatic cancer. The results showed that MTV and MTV/ADCmin were significantly higher in T4, N1, M1 and TNM stage 3+ stage tumors.

In addition, compared with MTV and ADCmin, the area under the receiver operating characteristic (ROC) curve (was the highest, and the above studies showed that the imaging parameters of PET/MRI could predict the clinical stage of pancreatic cancer. Joo (37) et al. showed that in terms of preoperative staging (N and M staging) and resectability evaluation of pancreatic tumors, the AUC of PET/MRI and PET/CT were 0.891 vs 0.776; N staging accuracy was 54% vs 31%,; and M staging accuracy: was 94% vs 88%, demonstrating no significant differences; however, PET/MRI produces lower radiation. Furtado (38) explored the effect of 37 PET/MRI results of 25 patients on the change in treatment strategy, and found that 18/37 (49%) of PET/MRI results altered the treatment strategy of patients. This study also affirmed the importance of PET/MRI in initial patient staging and treatment decisions. Integrated PET/MRI imaging can play a valuable role in shortening the preparation time of suspected pancreatic tumors and avoiding the transition from resectable to unresectable state due to the aggressive biology of pancreatic tumors.

These studies have demonstrated the feasibility of clinical ^18^F-FDG PET/MR imaging but have not shown substantial improvements in staging. This situation may not be unexpected since MR imaging has not replaced CT for staging of the malignancies for which ^18^F-FDG PET/CT is most commonly used (1). Given the widespread concerns about rising health care costs in general and the costs of advanced imaging techniques in particular, establishing ^18^F-FDG PET/MRI for whole-body, cancer staging may be challenging because it requires more expensive equipment and longer acquisition times than ^18^F-FDG PET/CT. In conclusion, PET/MRI offers countless, potential advantages over PET/CT (e.g., lower radiation exposure, higher soft-tissue contrast, and multiparametric imaging). Realizing this potential in clinics will likely require novel radiopharmaceuticals and applications other than whole-body cancer staging.

### 4.4 The value of 18F-FDG PET/MRI in evaluating the efficacy of pancreatic cancer

Most pancreatic cancers are found in a state of borderline resectable, locally-advanced, or distant metastasis; neoadjuvant or palliative therapy is the first choice for their treatment. After neoadjuvant therapy or palliative therapy, accurate efficacy assessment is crucial for the timing of surgery and the selection of subsequent treatment options. However, conventional CT and MRI have certain limitations for early and accurate assessment of the treatment response of borderline resectable, locally-advanced, and metastatic pancreatic cancer. It has been shown (39) that changes in tumor size measured by CT underestimate the effectiveness of neoadjuvant therapy in borderline resectable pancreatic cancer according to Response Evaluation Criteria in Solid Tumors (RECIST). It is challenging to distinguish post-treatment fibrosis from surviving tumors when analyzing anatomical images thus reducing the accuracy of tumor resectability after neoadjuvant therapy. In pancreatic cancer with locally-advanced or distant metastases, tumor size change is a relatively late endpoint. As a result, many patients may miss out on surgery or are no longer eligible for second-line therapy while waiting for anatomical imaging to determine response to therapy. PET/MRI has the advantages of functional imaging of PET and multiparameter imaging such as DWI, which are expected to overcome the limitations of CT and MRI in evaluating treatment response, and achieve earlier and more effective monitoring of treatment response.

Panda (40) discussed the value of PET/MRI imaging parameters in the evaluation curative effects after neoadjuvant therapy for borderline resectable and locally-advanced pancreatic cancer. Complete metabolic response (CMR), ΔSUVmax, ΔSUVgluc were significantly higher than those that were ineffective, and both ΔSUVmax and ΔSUVgluc used a cutoff value of 64% to distinguish effective and ineffective patients. This study demonstrates that metabolic markers of PET/MRI can be used to evaluate the severity of borderline resectable and locally-advanced pancreatic cancer after neoadjuvant therapy and then guide treatment decisions after neoadjuvant therapy. Wang (41) et al. explored the differences in PET/MRI imaging parameters of 13 patients with locally-advanced and metastatic pancreatic cancers before and four weeks after treatment. MTV, TLG decreased values ​​and ADCmean, ADCmin increased values ​​were significantly higher than in ineffective treatment. These findings suggest that PET/MRI can provide early treatment response assessment in patients with locally-advanced and metastatic pancreatic cancers.

18F-FDG PET/MRI predicts the chemotherapy effect after two weeks of neoadjuvant or palliative chemotherapy in pancreatic ductal adenocarcinoma patients (42), and the results show that two weeks after chemotherapy initiation, ΔMTV50%, ΔADC and ΔTLG50%, make it possible to predict responders and non-responders, respectively. A combination of parameters (i.e., ΔTLG50% and ΔADCmax or ΔMTV50% and ΔADCmax) further improved discrimination. Multiparameter 18F-FDG PET/MRI-derived parameters, particularly indicators of tumor glycolysis and cytoarchitectural changes, enable very early prediction of chemotherapy response.

Therefore, the imaging parameters of PET/MRI are expected to minimize the toxicity of ineffective treatment and guide the clinical adjustments of treatment plans for in operable patients. It was found that FDG PET/CT plus delayed PET/MR imaging, the diagnostic performance in the assessment of tumor resectability was not significantly different from CE-CT/MRI imaging, and the area under the receiver operating characteristic curve was 0.927 vs 0.925.The N staging accuracy was 80% vs. 55%, and the M staging accuracy was 100% vs. 93% (43). Among the 14 patients with liver metastases, PET/MRI detected the largest number. It was confirmed that FDG PET/CT plus delayed PET/MRI imaging showed similar diagnostic performance to CE-CT/MRI in the pretreatment assessment of pancreatic tumor resectability and staging, and more importantly, delayed PET/MR imaging provided increased metastatic information.

Delta Radiomics Analysis for Local Control Prediction in Pancreatic Cancer Patients Treated Using Magnetic Resonance Guided Radiotherapy (44). The result showed that Delta Radiomics analysis on low-field MR images might play a promising role in 1yLC prediction for LAPC patients. In addition, Delta radiomics analysis of Magnetic Resonance guided radiotherapy imaging data can enable treatment response prediction in pancreatic cancer (45). The research also confirmed the value of MRI radiomics in the evaluation of the treatment effect of pancreatic cancer

### 4.5 The value of 18F-FDG PET/MRI in assessing prognosis and monitoring recurrence of pancreatic cancer

Although the treatment of pancreatic cancer has progressed in recent years, the prognosis is still poor. Therefore, prognostic evaluation is highly critical in diagnosing and treating pancreatic cancer. Chen (46) et al. explored the correlation between imaging parameters (e.g. PET/MRI, dynamic contrast-enhanced MR, DWI, MRS and overall survival (OS)) of pancreatic cancer patients. The results showed that patients with low TNM stage, high peak value, high ADCmin value and low TLG value had better OS. Furthermore, a high TLG/peak ratio was strongly associated with low OS, and in a group of patients who did not undergo radical surgery, the TLG/peak ratio was an independent predictor of OS. Additionally, the MTV/ADCmin ratio was an independent predictor of progression-free survival (PFS) after adjusting for age, sex, tumor size, and stage (36). Dunet et al. (47) also explored the prognostic values of PET and MRI imaging parameters for OS, disease specific survival (DSS) and PFS in untreated pancreatic cancer patients. The results showed that MTV is an independent predictor of OS and DSS, the total volume of diffusion was an independent predictor of PFS.

In prognostic evaluation of pancreatic cancer patients with local progression and metastasis after four weeks of chemotherapy, taking PET MTV reduction of 60% or TLG reduction of 65% as the threshold, MRI ADCmean or ADCmin value increased the threshold by 20%, indicating the effective rate is higher than the ineffective or has longer PFS and OS. Panda (40) compared borderline resectable and locally-advanced pancreatic cancer after neoadjuvant therapy and found that CMR, ΔSUVmax, and ΔSUVgluc were closely related to OS. In addition, comparing the metabolites of 1H-MRS in pancreatic cancer and normal pancreases was associated with PET metabolic activity, clinical stage and survival outcomes; it was additionally discovered that high TNM stage, low creatine, low glutamine (Glx) and low blood lipid levels were negatively correlated with PFS and OS (all p<0.05). Glx, Cr and lipid levels were all negatively correlated with MTV and TLG of PET, and NAA was weakly and negatively correlated with SUVmax (48). A possible reason for the stronger correlation of MRS metabolites with MTV and TLG rather than SUVmax is that MRS, MTV, and TLG are all volumetric parameters whereas SUVmax only represents the single, highest metabolic pixel value of the tumor. The above-mentioned studies have shown that the multiple imaging parameters of PET/MRI are beneficial for the evaluation of the prognosis of pancreatic cancer patients before and after treatment.

Recurrence of pancreatic cancer after surgical resection is common, and early detection of recurrence is critical for treatment decisions. However, postoperative changes can simulate or mask tumor recurrence, so standard CT and MRI may not be sufficiently accurate. Dalah et al. (49) found that 15% of patients had progressive disease when using Positron Emission Tomography Response Criteria in Solid Tumors (PERCIST1.0)criteria, as opposed to only 7% when they applied response evaluation criteria in solid tumors version 1.1 (RECIST1.1) using CT or CE-CT images. Panda et al. (40) lso showed that hybrid PET/MRI can help ascertain pathological response to therapy in PDAC with a high negative predictive value. In addition, available data indicate that FDG PET/MRI can be useful for predicting and assessing response to CRT in patients with resectable or borderline PDAC (39).

While PET/CT is functional imaging, PET/CT shows higher sensitivity than CT or MRI in detecting recurrence. However, fibrosis due to chemotherapy or radiotherapy or changes in the local anatomy after surgery can also affect the assessment of PET/CT to some extent, especially in the complex retroperitoneal space where the lesions of FDG uptake is difficult to assess. Integrated PET/MRI has an excellent, spatial colocalization ability; combined with its superior soft-tissue contrast of MRI, it is conceivable that PET/MRI can overcome the complexity of postoperative, anatomical structure to a certain extent and improve the detection rate of recurrent lesions. Of course, more research is needed in the future to confirm this speculation.

With the successful development and clinical application of the integrated PET/MRI imaging system, its advantages have gradually emerged in the detection, initial staging, preoperative evaluation, efficacy evaluation, prognosis evaluation and monitoring of recurrence of pancreatic cancer. Some of the research results are shown in **Table 1**. More PET/MRI-based clinical trials are underway, and as these studies are completed, the value of PET/MRI in the clinical management of pancreatic cancer will be more fully demonstrated and realized (**Table 2**).

**Table 1:** Summary of studies related to the application of PET/MRII in pancreatic cancer.

Related research	Enrolled patients	research objective	PET/MRI technology (integrated/heterogeneous fusion; enhanced/plain scan)	Contrast image	Main research results
Chen et al	63 cases of pancreatic cancer	Prognosis evaluation	integrated; enhance	/	The TLG/peak ratio reflects a mismatch of blood flow in pancreatic cancer patients, which may better predict OS than other imaging biomarkers of PET/MRI.
Dunet et al	61 patients diagnosed with pancreatic cancer	Prognosis evaluation	Different machine fusion; plain scan	/	MTV is an independent predictor of OS and DSS, and DTV is an independent predictor of PFS
Panda A	44 patients with borderline resectable or locally advanced PDAC underwent PET/MRI before and after treatment	Pathological response to NAT and predict of OS	integrated;	Enhanced CT	After NAT, the changes of metabolic indexes from PET/MRI and morphological indexes from CT were related to the pathological reaction and OS of PDAC patients.
Chen et al	60 patients with pancreatic cancer or periampullary cancer	Clinical stage and prognosis	Integrated PET/MRI system		PFS PET/MRI imaging biomarkers can predict the clinical stage and PFS of patients with pancreatic cancer or periampullary carcinoma.
Wang et al.	(PDAC)13 patients with PDAC	Treatment evaluation	Integrated	/	PET/MRI can evaluate the early response of patients with advanced PDAC, so it is possible to make non responders adapt to treatment early.
J Ruf	32 (15 pancreatic cancer and 17 benign diseases).	diagnosis	Fusion image		PET/MRI image fusion improves the anatomical assignment and interpretation of FDG lesions. However, for patients with multiple lesions or primary incurable diseases, the therapeutic effect is limited.
Tatsumi et al.	47 patients with pancreatic cancer	Diagnosis and differential diagnosis	Fusion image	CT/MRI	PET/MRI fusion, especially PET and T1WI MRI, is superior to PET/CT in characterizing Pt
Sagheb	10 patients with pancreatic cystic tumors	Diagnosis and differential diagnosis	Integrated	/	PET/MRI can be used to distinguish SCN from non SCN lesions in pancreatic serous cystic tumors.

**Table 2:** Clinical trial of pancreatic lesions based on PET/MRI.

Study type	Title of trail	Aim of trail	Number of patients (n)
NCT03352037	18F-FDG PET/MR Imagingfor Differentiation of Serous From Non-Serous Pancreatic Cystic Neoplasms: A Pilot Study	to evaluate if integrated 18F-FDG PET/MR imaging with simultaneous MR and PET acquisition is helpful in differentiation of SCN from nonserous lesions.	11
NCT02550847	Evaluation of Prognosis With Integrated MRI/PET in Patients With Pancreatic Cancer	to evaluate MR/PET with both functional and molecular imaging on the prediction of prognosis in patients with suspected pancreatic cancer.	100
NCT04158414	Applying PET/MR in Oncology - a Prospective Project	Early detection of cancer through screening based on imaging is probably a major contributor to a reduction in mortality for certain cancers.	500
NCT04395469	FAZA PET/MRI Pancreas	This study will assess whether or not PET/MRI scans can provide useful information about hypoxia in pancreatic cancer.	20
NCT03918759	Diagnostic Accuracy of Preoperative Diagnostic Procedure in the Assessment of Lymph Node Metastases by NF-PanNENs	In this study the investigators will evaluate prospectively the accuracy of these diagnostic exams in detecting the lymph node status. Patients with sporadic NF-PanNEN who are candidates for surgical resection will undergo CE-CT scan, 68Ga DOTATOC (and eventually 18F-FDG) PET/MRI and EUS with FNA/B.	150
NCT02500004	Brown Adipose Tissue Activity and Energy Metabolism in Cachexia (BAT-Cachexia)	determine BAT activity in cachectic patients with pancreatic or non-small cell lung cancer, and in cachectic patients with chronic obstructive pulmonary disease (COPD), and compare results with healthy individuals and non-cachectic COPD patients, matched for age and BMI.	16

### 4.6 The value of 18F-FDG PET/MRI in other types of pancreatic malignancies

Nonductal epithelial malignancies of the pancreas include pancreatic blastoma and pancreatic neuroendocrine neoplasms (PanNEN). The various malignant pancreatic lesions have different PET/MRI appearances. The role of PanNEN risk stratification was assessed using 68Ga-DOTATOC and 18F-FDG combined with PET/CT and PET/MRI-derived parameters to determine the imaging profiles that influence the risk of patients undergoing PanNEN surgery, particularly with regard to histological features of invasiveness (50). It was found that 68Ga-DOTATOC SUVmax significantly predicted distant metastasis with a threshold of 51.27 (sensitivity and specificity were 85.7% and 68.1%, respectively). When the cutoff threshold for the parameter MTV for 18F-FDGPET was 7.98 (sensitivity and specificity were 69.7% and 82.4%, respectively), the cutoff threshold for TLG was 32.4 (sensitivity and specificity were 69.7% and 82.4%, respectively). MTV and TLG were predictors of vascular invasion. Dual tracer 68Ga-DOTATOC and 18F-FDG PET scans can provide relevant information on tumor-based biological behavior and invasiveness, and provide a reference for accurate staging.

Ductal epithelial malignancies of the pancreas also include intraductal papillary mucinous tumors, and 18F-FDG PET/MRI can be used to assess whether they have high, malignant potential (31). A 57-year-old man underwent 18F-FDG PET/CT and PET/MRI to evaluate a pancreatic head mass. The low-dose non-enhanced CT portion could not identify the exact location of metabolic abnormalities on PET; but PET obtained 60 minutes after the end of the PET/CT scan in the PET/MRI image, it was found that the section with increased metabolism was located on the slightly enhanced cyst wall, which to some extent suggested that the intraductal papillary mucinous tumor of the pancreas has a certain malignant tendency. Finally, it was confirmed by pathology that the increased metabolism on PET was the columnar tumor with mucin-filled dilation and cell arrangement on the distorted pancreatic duct. This case shows that comparing with 18F-FDG PET/CT, 18F-FDG PET/MRI can improve the detection ability of some uncertain and accidental lesions in the pancreas.

## 5 Novel, positron-imaging agents assists PET/MRI in the diagnosis of malignant, pancreatic lesions

Pancreatic ductal carcinoma has a raised mortality rate, and early detection and accurate staging are critical for prolonging survival. With the significant advantages and potential of PET/MRI in malignant, pancreatic lesions, an abundance of new, positron-imaging agents have emerged. For example, 68Ga-labeled fibroblast activation protein inhibitor (68Ga-FAPI) has gradually shown unique value in tumor PET imaging of malignant lesions, including that of pancreatic lesions. Zhang et al. (51) conducted imaging follow-up or pathological results of lesions with 68Ga-FAPI-04 uptake, and the results showed that 68Ga-FAPI-04 uptake can occur in any part of the pancreas. When the lesions are small, there may be no apparent changes on CT or MRI, but 68Ga-FAPI-04 PET/MRI can determine the either benign or malignant nature of these lesions with uptake. Although the lesions identified in this study were all benign, 68Ga-FAPI-04 PET/MRI could increase the accuracy of our diagnosis of benign, pancreatic lesions. In addition, 68Ga-DOTATOC PET/MRI imaging and radiomic parameters can predict histopathological, prognostic factors in patients undergoing PanNET surgery (52). Continuous 68Ga-DOTATOC PET/MRI scans of PanNETs were used to obtain SUVmax, SUVmean, somatostatin receptor density (SRD), total lesion somatostatin receptor density (TLSRD) and MRI ADC, and arterial and late enhancement, Data such as necrosis, cystic degeneration, and maximum diameter were then extracted from PET and MRI scans for first-, second-, and higher-order radiomic parameters. Finally, SUVmax and SUVmean were dependable predictors of lymph node (LN) involvement (AUC 0.850 and 0.783, respectively). The second-order radiomic parameters GrayLevelVariance and HighGrayLevelZoneEmphasis extracted from T2MRI showed a significant correlation with LN involvement and also showed good predictive performance (AUC = 0.992). Demonstrating the synergistic effect of PET/MRI on the imaging parameters extracted by the two modalities, the potential of PET imaging and radiological parameters in assessing the histopathological features of PanNET invasiveness is enormous.

In addition, the uptake of 18F-FDG, 3-deoxy-3-18F-fluorothymidine (18F-FLT), and 18F-fluoroethylcholine (18F-FEC) in human pancreatic tumor cell lines was assessed by PET/MRI. In a case study (39), 4 weeks after orthotopic inoculation of human pancreatic cancer cells (PancTuI, Colo357, and BxPC3) into SCID mice, 18F-FLT showed the highest tumor uptake with a mean TLR of 2.3, which correctly displayed all 12 pancreatic tumors. 18F-FDG detected only 4 out of 8 tumors, and uptake was low, with a mean TLR in tumors of 1.1. 18F-FEC did not show any tumor uptake. Gene array analysis revealed that the rate-limiting enzyme hexokinase and pancreatic-specific glucose transporter 2 were significantly downregulated by 18F-FDG compared with normal, pancreatic duct cells and pancreatic tumor tissues.

In contrast, thymidine, which is responsible for the capture of 18F-FLT, was significantly downregulated. Kinase 1 was significantly upregulated in tumor cell lines, whereas genes involved in 18F-FEC uptake were not affected nor downregulated in tumor cell lines. The findings demonstrate that 18F-FLT as a PET tracer has the highest and most consistent uptake in various human, pancreatic tumor cell lines in SCID mice compared to 18F-FDG and 18F-FEC. Different tracers can reflect the uptake ability of pancreatic tumor cells through different PET uptake values.

Nielsen (53) et al. used 64Cu-labeled active site inhibitor factor VII for PET imaging of pancreatic cancer tissue factor as tissue factor (TF) is the main initiator of the extrinsic, coagulation cascade and plays an important role in tumorigenesis. TF expression has been reported in 53%-89% of pancreatic cancers, and TF expression levels in clinical studies have been associated with later staging, increased microvessel density, increased metastasis, and poorer OS. Imaging TF as a prognostic biomarker and TF-directed therapies currently in clinical development have clinical relevance. In this study, *in vivo* PET imaging was performed 1, 4, 15 and 36 hours after injection of 64Cu-NOTA-FVIIai in pancreatic adenocarcinoma (BxPC-3) mice. The *in-vitro* experiments were compared *via* flow cytometry and immunohistochemistry. Increased tumor-to-normal tissue contrast was ultimately observed; tumor uptake of unlabeled nuclides was significantly reduced for 64Cu-labeled FVIIai compared to unlabeled FVIIai. Tumor uptake was significantly different with variable TF expression levels and was consistent with TF levels assessed *via* immunohistochemical staining. Tumors *in situ* were also clearly visible on PET/MRI images and were consistent with uptake matches for 64Cu-NOTA-FVIIai. This shows that 64Cu-NOTA-FVIIai is very suitable for PET imaging of tumor TF expression, and the TF expression level of different pancreatic tumor models can be distinguished with the use of PET/MRI.

There is increasing evidence that neurotensin (NT) and neurotensin receptors (NTR) play critical roles in the growth and survival of pancreatic neoplasms. Small animal PET scans performed following administration of [18F] AlF-NOTA-NT found that this probe had high contrast between that tumor and background at that 1 and 4 hr time points in NTR1-positive pancreatic tumor models (AsPC-1 and PANC-1). In blocking experiments 4 hours post-injection, the pancreatic cancer model with the blocker (unlabeled NT probe) exhibited significantly higher tumor uptake than the model without the blocker (ID/g 1.0 ± 0.2 vs 0.1 ± 0.0%) (54). In addition, when NTR1 was labeled with imaging probes synthesized from 64 Cu-AmBaSar-NT and IRDye800-NT *in vivo (* 55), *in-vivo* imaging showed higher uptake in 64 Cu-AmBaSar-NT tumors; one h and four h post-injection ID/g were 3.76 ± 1.45 and 2.29 ± 0.10%, respectively; fluorescence imaging of IRDye800-NT showed good, tumor contrast. The above results indicate that NTR1 is a promising target for imaging and treatment of pancreatic ductal adenocarcinoma. It is expected that patients receiving NTR-targeted therapy will be screened and monitored for efficacy in the future to realize personalized medicine.

## 6 Novel, MRI-contrast agents enable PET/MRI diagnosis of pancreatic lesions

Contrast-enhanced MRI is most commonly used in the differentiation of benign versus malignant pancreatic growths, especially in the arterial phase, which has a high, diagnostic performance. However, diagnosis of pancreatic cancer *via* MRI requires contrast agents with higher specificity and sensitivity. A recent study (56) used small heat shock protein 16.5-based nanocages and gadolinium(III)-chelated contrast agents in a KPC, genetically engineered mouse model. With the iRGD peptide (targeting neuropilin-1 expressed on pancreatic cancer cells), MRI can detect neuropeptide-1 positive cells expressed on pancreatic cancer cells as well as significantly-enhanced signal of pancreatic cancer. This indicates that the novel, iRGD-modified nanocages, as a specific and sensitive MRI-contrast agent, have potential application value in the diagnosis and clinical application of pancreatic tumors.

Erica Locatelli (57) et al. developed a novel multifunctional biocompatible hybrid nanocarrier for PET/MRI dual imaging, and organic ligand-encapsulated synthetic hydrophilic superparamagnetic maghemite nanoparticles (NPs) It can not only be used as a contrast agent for MRI but also has a 68Ga-chelating group on its outer surface, which is also used as a labeling drug for *in-vivo* PET imaging, making the NP-NODA-68Ga complex more stable. Cytotoxicity assays found that Magh-1-PNPs-NODA was not toxic to seven PDAC cell lines *in vitro*. Their results suggest that the new nanocarriers are promising tools for the future development of PET/MRI diagnostic reagents.

## 7 Summary and outlook

In conclusion, PET/MRI combines the advantages of PET’s highly-sensitive metabolic and functional imaging with MRI’s increased, soft-tissue contrast and multisequence-multiparameter imaging. In addition to being conducive to develop individualized treatment plans, it can also facilitate evaluative efficacy and accurate prognosis. In addition, integrated PET/MRI can develop new biomarkers, providing a unique and potential research direction for future pancreatic cancer research. Interdisciplinary fields represented by radiomics and artificial intelligence, have expanded new horizons for PET/MRI research, represented by newfound PET-imaging agents and novel, MRI-contrast agents, deepening the application of PET/MRI in the comprehensive management of pancreatic cancer. However, the current research still faces problems, such as a small number of samples; some are allogeneic fusions. Research reports on the application of prospective, large-sample, integrated PET/MRI in pancreatic cancer are still limited, and more research is needed to confirm their findings in the future. The value of PET/MRI in diagnosing and treating pancreatic cancer is gratifying because several, clinical trials are being carried out in an orderly manner (**Table 2**). With the gradual disclosure of the results of these studies, the value of PET/MRI in the clinical management of pancreatic cancer will become more fully understood and provide safer, more efficacious services for the majority of pancreatic cancer patients.

## Data availability statement

The datasets generated during and/or analyzed during the current study are available from the corresponding author on reasonable request.

## Author contributions

JL: original draft. CF: review and editing. SZ: methodology. YP: visualization, investigation. FY: supervision. SGZ: software, validation. CY: funding acquisition. HG: project administration. LC: writing- reviewing and editing. All authors contributed to the article and approved the submitted version.
